# Assessing global drivers of parasite diversity: host diversity and body mass boost avian haemosporidian diversity

**DOI:** 10.1017/S0031182024000313

**Published:** 2024-04

**Authors:** Daniela de Angeli Dutra

**Affiliations:** Department of Zoology, University of Otago, PO Box 56, Dunedin, New Zealand

**Keywords:** avian malaria, functional diversity, haemosporidian, host diversity, migration, parasite diversity

## Abstract

Biodiversity varies worldwide and is influenced by multiple factors, such as environmental stability and past historical events (e.g. Panama Isthmus). At the same time, organisms with unique life histories (e.g. parasites) are subject to unique selective pressures that structure their diversity patterns. Parasites represent one of the most successful life strategies, impacting, directly and indirectly, ecosystems by cascading effects on host fitness and survival. Here, I focused on a highly diverse, prevalent and cosmopolitan group of parasites (avian haemosporidians) to investigate the main drivers (e.g. host and environmental features) of regional parasite diversity on a global scale. To do so, I compiled data from 4 global datasets on (i) avian haemosporidian (malaria and malaria-like) parasites, (ii) bird species diversity, (iii) avian functional traits and (iv) climate data. Then, using generalized least square models, I evaluated the effect of host and environmental features on haemosporidian diversity. I found that haemosporidian diversity mirrors host regional diversity and that higher host body mass increases haemosporidian diversity. On the other hand, climatic conditions had no effect on haemosporidian diversity in any model. When evaluating *Leucocytozoon* parasites separately, I found parasite diversity was boosted by a higher proportion of migratory hosts. In conclusion, I demonstrated that haemosporidian parasite diversity is intrinsically associated with their hosts’ diversity and body mass.

## Graphical abstract

**Figure:**
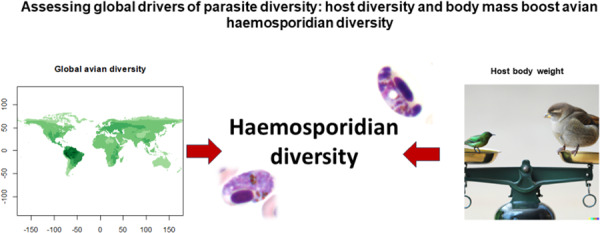

## Introduction

Variation in global biodiversity is ruled by several historical and ecological factors, such as environmental stability and productivity and major geographical events (e.g. the formation of the Panama Isthmus a few million years ago). For instance, regions thought to be more productive and stable through evolutionary time harbour greater biodiversity (e.g. neotropics) (Rull, 2011). Increases in environmental productivity and stability could promote greater niche partitioning, thus enhancing species diversification, and as a result, expanding regional biodiversity (Rull, 2011; Burin *et al.* 2021). However, the exact mechanisms that promote increases in biodiversity are still not well understood. Nevertheless, the drivers of biodiversity should be intrinsically associated with their life histories and strategies. For instance, since parasites extract their resources from their hosts, these organisms require the presence of competent hosts to colonize and/or thrive in certain regions (Mestre *et al.* 2020). At the same time, internal parasites and other symbionts are only indirectly affected by climatic conditions since they are often not directly exposed to the environment. Hence, parasite/symbiont diversity is subject to specific evolutionary and ecological pressures that can differ from those affecting free-living organisms.

Host biodiversity has been identified as one of the main predictors of parasite diversity (Kamiya *et al*., 2014a, 2014b; Martins *et al*., 2020). Indeed, host biodiversity can enhance parasite diversity by (i) increasing colonization options (more species available), (ii) segregating parasite species populations and, (iii) supporting a greater variety of parasite life cycles (Hechinger and Lafferty, 2005). In addition, since parasites can coevolve with their hosts (Park *et al*., 2020; de Angeli Dutra *et al*., 2022*a*), host diversification events might promote parasite speciation due to the niche partitioning process (i.e. parasite specialization into a single new host species). Furthermore, host functional traits can directly affect parasite life cycles and, consequently, promote or reduce diversification. For instance, heavy-bodied hosts harbour higher parasite diversity (Kamiya *et al*., 2014*a*). Migratory behaviour provides an opportunity for parasites to reach new regions of the globe, expanding their geographical and host range (de Angeli Dutra *et al*., 2021; Poulin and de Angeli Dutra, 2021). On the other hand, the resident host fauna can also enhance parasite diversity by providing a stable resource. Meanwhile, territoriality may reduce interactions among species. As a result, resident and territorial fauna could enable greater diversification *via* niche partitioning processes and speciation.

Environmental features also shape species diversification by driving (i) regional productivity and ecosystem energy levels, (ii) biological tolerance levels (i.e. harsher environments tend to present lower levels of biodiversity) and (iii) ecological stability over evolutionary time. The latter (i.e. ecological stability) can enhance diversification because stable environments allow species to specialize in particular resources, increasing the availability of vacant niches and, consequently, increasing opportunities to diversify into new species. Naturally, certain environmental conditions are more likely to result in species diversification than others. For instance, diversity is concentrated in the tropics (i.e. more productive and stable regions), a trend known as the Latitudinal Diversity Gradient (Hillebrand, 2004; Rull, 2011). Environmental features can directly or indirectly affect parasite life cycles depending on their life strategy (de Angeli Dutra *et al*., 2022*b*). Vector-borne parasite distribution is often associated with climate conditions, due to, for example, thermal constraints in parasite development (Lapointe *et al*., 2010; Mordecai *et al*., 2013). Hence, environments that offer better conditions for vector development (e.g. high temperature and precipitation) are expected to harbour greater prevalence and diversity of vector-borne parasites (McNew *et al*., 2021; Fecchio *et al*., 2021*b*). Likewise, since vectors are ectothermic organisms, the external temperature might directly shape parasite development, transmission, and, as a result, diversity.

Avian malaria and malaria-like (haemosporidian) parasites are cosmopolitan protozoan vector-borne parasites transmitted by dipterans (Valkiūnas, 2005). They are mainly represented by 3 distinct genera: *Plasmodium*, *Haemoproteus* and *Leucocytozoon*. Avian haemosporidians are among the most prevalent and diverse avian parasites, comprising more than 300 distinct species and 4000 unique parasite lineages (Valkiūnas, 2005; Bensch *et al*., 2009). Due to the relevance of vector-borne diseases to human health, these parasites are frequently used as ecological models of host–parasite interactions. Previous studies have culminated in an online global database on avian malaria and malaria-like parasites established in 2009 and updated ever since (MalAvi http://130.235.244.92/Malavi/, Bensch *et al*., 2009). In addition, information regarding their hosts' (i.e. birds) distribution, biodiversity and functional traits is extensive and easily available online. Hence, avian haemosporidians represent the ideal model system to investigate the drivers of parasite diversity worldwide.

Previous research on haemosporidians has also accessed drivers of haemosporidians on a global scale (Clark *et al*., 2014; Clark, 2018), evaluating the role of host hot spots, latitude and climate. Here, I evaluated for the first time the effect of host phylogenetic diversity and functional traits (e.g. territoriality, migratory status, range size and body mass) on haemosporidian phylogenetic diversity at a global scale. Like former research, I have also included climatic conditions (i.e. temperature and precipitation patterns) in my analyses. Here, I predicted that (i) bird phylogenetic diversity and functional traits drive parasite diversity and (ii) haemosporidian diversity increases with higher temperature and precipitation rates. My goal was to uncover the main drivers of avian haemosporidian diversity.

## Methods

### Dataset

**Figure 1.**
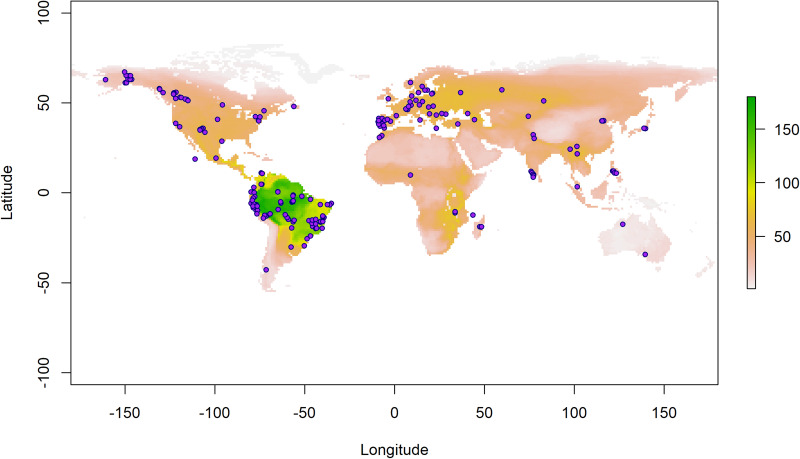
Bird collection sites. The colour scale represents spatial variation in bird species richness worldwide. Collection sites comprise a total of 100 regions and 207 localities (including offshore islands) extracted from the MalAvi database.

I obtained data from 4 open online datasets to conduct this research. Firstly, the MalAvi (http://130.235.244.92/Malavi/) (Bensch *et al*., 2009) database was used to extract data on haemosporidian (i.e. *Plasmodium*, *Haemoproteus* and *Leucocytozoon*) using the function ‘extract_table’ from the ‘malaviR’ package in R in November 2021 (R Core Team, 2017). MalAvi contains records of haemosporidian parasites for each site sampled. Here, however, I excluded from the analyses all sites with fewer than 10 records (Fig. 1). Bird distribution polygon format files were acquired from BirdLife International (https://www.birdlife.org/) (BirdLife International and Handbook of the Birds of the World (2020) Bird species distribution maps of the world. Version 2020.1. Available at http://datazone.birdlife.org/species/requestdis.).

Bird functional traits (i.e. body mass, range size and territoriality) data were extracted from Open Traits datasets (https://opentraits.org/datasets.html) (Wilman *et al*., 2014). To classify birds into migratory categories (e.g. resident and migratory), I used data published by Dufour *et al*., 2020. Body mass and range size represent quantitative variables, while migratory status and territoriality are categorical variables. Lastly, climatic data (i.e. temperature and precipitation conditions) was extracted from Wordclim (https://worldclim.org/) using the function ‘getData’ from the ‘raster’ package in R and resolution equal to 10 km. Climatic data here consisted of 19 distinct quantitative climatic features relating to temperature and precipitation measures. Due to the high correlation among several predictors (Supplementary Figs 1 and 2), I only kept host body mass and migratory distance as functional host trait variables and 4 climatic metrics (mean annual value and seasonality in both temperature and precipitation) in my analyses. Those metrics were chosen because they represent a metric of annual mean values and their variation (i.e. seasonality).

Since I compared values among distinct areas of the globe, data was clustered into regions based on their geographic coordinates using geographic cell grids of 5 × 5 degrees to calculate both host and parasite phylogenetic diversity. Those grids were treated as distinct geographical units, each characterized by the occurrence of particular haemosporidian lineages, bird species, and their traits, and environmental conditions. Overall, the final dataset consisted of geographical grid ID, the regional parasite and host phylogenetic diversity, and the respective mean information on regional climate conditions and mean host body mass. For migratory behaviour, I created a dummy table separating each migratory status in a different column and calculated the percentage of migrants in each quadrant.

### Calculating parasite diversity

Parasite diversity was calculated at the level of each geographical coordinate grid. To estimate haemosporidian regional diversity (alpha-diversity), I created a phylogenetic tree for haemosporidian parasites. Here, I included 2016 parasite lineages extracted from the MalAvi dataset using the ‘long sequences’ data (i.e. complete sequences only). JmodelTest (Posada and Crandall, 1998) and Mr. Bayes (Ronquist and Huelsenbeck, 2003) were implemented for model selection and Bayesian tree compilation, respectively. The haemosporidian phylogeny was built following inverse-gamma substitution rate distribution, 25% burn-in. A total of 50 000 000 iterations, 4 chains, and 2 runs were performed using CIPRES with printing and sampling frequencies set at 1000 (Miller *et al*., 2015). A decision criterion was included based on a posterior probability greater than 0.01. Subsequently, ‘sump’ and ‘sumt’ commands were used to summarize parameter values and to produce a consensus tree. Convergence was assessed every 5000 generations. The final haemosporidian phylogeny was used to calculate parasite diversity considering phylogenetic differences among parasites inhabiting a region. To do so, I calculated Faith's Phylogenetic Diversity, which calculates the sum of the total phylogenetic branch length for one or multiple samples (i.e. genetic distances) (Kembel *et al*., 2010). Analyses were repeated for each genus using cropped phylogenetic trees containing branches for each genus individually.

### Calculating host diversity

Since the phylogenetic relationships among hosts are a substantial factor driving haemosporidian assemblages (Lutz *et al*., 2015; Aguiar de Souza Penha *et al*., 2022; De La Torre *et al*., 2022), we calculated host diversity using a metric that considers the phylogenetic distances among hosts. We can use Hill numbers to normalize diversities and compare diversity among regions in a more intuitive manner. Hill numbers represent the effective number of species or phylogenetic entities in an assemblage. For this reason, I used phylogenetic hill numbers to calculate host diversity in this study. Using a full avian phylogeny file from the AllBirdsHackett1.tre website (https://birdtree.org/) (Jetz *et al*., 2012) and used the ‘treeman’ package (Bennett *et al*., 2017) to create a file containing all trees from the original file. Then, I randomly selected a phylogenetic tree as the creation of a consensus tree branch lengths, which are used to calculate phylogenetic diversity. Species not found in our data were excluded from the host phylogenetic tree. An occurrence matrix was then created to assign the presence of each bird species to the geographic grids in which they were found. Finally, we calculated phylogenetic hill numbers to estimate host diversity using the occurrence and phylogenetic data.

### Statistical analyses

All analyses were run in R (R Core Team, 2017). Due to the high spatial correlation of our data (Moran *I* value = 0.56), generalized least square models (GLSMs) were run to evaluate the drivers of haemosporidian diversity. I considered regional phylogenetic parasite diversity as a response (i.e. phylogenetic diversity of parasites in each quadrant) and bird body mass and migratory status (i.e. percentage of migrants in each quadrant), bird phylogenetic diversity, climatic conditions (i.e. mean temperature and precipitation and their seasonality), and sampling effort (i.e. the number of times haemosporidians were recorded in a region) as explanatory variables. Due to spatial correlation (Moran *I* = 0.56), I set both longitude and latitude as correlation variables in the models to account for non-independence among coordinate grids. The data was scaled (i.e. variable values represent standard deviations from the mean) before running GLSMs to account for metric variability in this study. I ran 4 models in total: one for all parasite genera combined and one for each parasite genus separately (i.e. *Plasmodium* lineages only, *Haemoproteus* lineages only and *Leucocytozoon* lineages only). It is important to note that MalAvi does not distinguish between *Haemopeoteus* and *Parahaemoproteus* parasites, therefore, both taxa were analysed using a single model containing all *Haemoproteus* lineages. Models' residuals were posteriorly checked to ensure model fitting.

## Results

**Figure 2.**
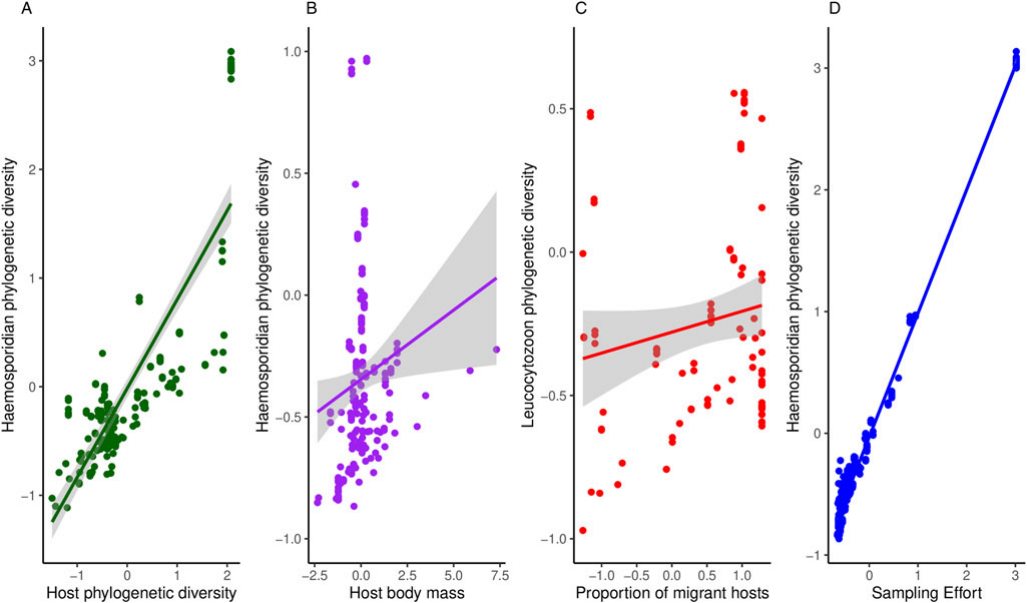
Relationship between phylogenetic haemosporidian diversity and A – host diversity, B – host body mass, C – the proportion of migratory hosts on *Leucocytzoon* diversity and D – sampling effort.

Haemosporidian taxonomic diversity ranged from 5 to 276 (in South America) distinct lineages per geographic region (i.e. defined as a geographic cell grid of 5 × 5 degrees, *n* = 100) and the highest number of parasite records in a region was 934. Overall, I found a mean parasite lineage diversity of 36 per region and an average of 71 records per region. The richness of bird species varied between 4 and 349, with a mean of 97 bird species per geographical region. In my models, I observed that haemosporidian phylogenetic diversity increased with host diversity and heavier-bodied hosts (Table 1, Fig. 2). The percentage of migratory hosts had no effect on overall diversity. Contrary to my hypothesis, climatic conditions had no effect on parasite phylogenetic diversity (Table 1).

**Table 1.** Estimates, standards error, confidence intervals and *P* values for host migratory status and body mass, climatic conditions, host diversity and sampling effort effects on phylogenetic haemosporidian diversity

Parameters	Estimates	Stand. error	Conf. int. (95%)		*P* value
Intercept	−0.04	0.04	−0.11	0.04	0.326
Migrants	−0.01	0.03	−0.05	0.07	0.760
**Body mass**	**0**.**06**	**0**.**02**	**0**.**03**	**0**.**10**	**<0**.**001**
Mean temp.	−0.02	0.03	−0.09	0.04	0.485
Temp. seasonality	−0.02	0.04	−0.11	0.06	0.581
Mean prec.	0.01	0.02	−0.03	0.06	0.541
Prec. seasonality	0.04	0.02	−0.01	0.08	0.106
**Bird diversity**	**0**.**09**	**0**.**05**	**0**.**00**	**0**.**19**	**0**.**05**
**Sampling effort**	**0**.**96**	**0**.**03**	**0**.**89**	**1**.**03**	**<0**.**001**

When analysing each parasite genus separately, I observed some differences among the different parasite genera. Host body mass was associated with parasite phylogenetic diversity in the models, except when evaluating *Plasmodium* parasites only. *Plasmodium* phylogenetic diversity was mostly driven by avian phylogenetic diversity, which boosts regional *Plasmodium* diversity (Table 2). Further, no host functional traits were associated with *Plasmodium* diversity. Both temperature and precipitation had no effect on *Plasmodium* diversity. *Haemoproteus* phylogenetic diversity seems driven only by host body mass. Neither migratory status nor climate variables had significant effects on *Haemoproteus* diversity (Table 3). Surprisingly, host phylogenetic diversity was not a predictor of *Haemoproteus* diversity. On the other hand, *Leucocytozoon* phylogenetic diversity increases with higher percentages of migratory hosts and heavy-bodied hosts in a region (Table 4). Again, contrary to my expectations, host phylogenetic diversity and both temperature and precipitation metrics were not associated with *Leucocytozoon* diversity. Sampling effort had the strongest effect, positively driving parasite diversity in all models.

**Table 2.** Estimates, standards error, confidence intervals and *P* values for host migratory status and body mass, climatic conditions, host diversity and sampling effort effects on phylogenetic *Plasmodium* diversity.

Parameters	Estimates	Stand. error	Conf. int. (95%)		*P* value
Intercept	0.02	0.08	−0.14	0.18	0.812
Migrants	0.11	0.07	−0.03	0.25	0.133
Body mass	0.05	0.04	−0.03	0.13	0.227
Mean temp.	0.08	0.07	−0.06	0.22	0.281
Temp. seasonality	−0.08	0.10	−0.27	0.12	0.423
Mean prec.	−0.006	0.05	−0.11	0.10	0.903
Prec. seasonality	0.02	0.05	−0.08	0.11	0.683
**Bird diversity**	**0**.**36**	**0**.**11**	**0**.**15**	**0**.**57**	**<0**.**001**
**Sampling effort**	**0**.**73**	**0**.**09**	**0**.**55**	**0**.**91**	**<0**.**001**

**Table 3.** Estimates, standards error, confidence intervals and *P* values for host migratory status and body mass, climatic conditions, host diversity and sampling effort effects on phylogenetic *Haemoproteus* diversity

Parameters	Estimates	Stand. error	Conf. int. (95%)		*P* value
Intercept	−0.06	0.04	−0.14	0.03	0.214
Migrants	0.01	0.04	−0.07	0.10	0.725
**Body mass**	**0**.**08**	**0**.**02**	**0**.**04**	**0**.**13**	**<0**.**001**
Mean temp.	0.06	0.04	−0.03	0.14	0.185
Temp. seasonality	−0.05	0.06	−0.16	0.06	0.393
Mean prec.	−0.006	0.03	−0.06	0.05	0.822
Prec. seasonality	0.02	0.03	−0.04	0.07	0.566
Bird diversity	0.07	0.06	−0.04	0.19	0.206
**Sampling effort**	**0**.**92**	**0**.**06**	**0**.**81**	**1**.**04**	**<0**.**001**

**Table 4.** Estimates, standards error, confidence intervals and *P* values for host migratory status and body mass, climatic conditions, host diversity and sampling effort effects on phylogenetic *Leucocytozoon* diversity

Parameters	Estimates	Stand. error	Conf. int. (95%)		*P* value
Intercept	−0.15	0.12	−0.39	0.09	0.219
**Migrants**	**0**.**25**	**0**.**04**	**0**.**17**	**0**.**32**	**<0**.**001**
**Body mass**	**0**.**14**	**0**.**02**	**0**.**10**	**0**.**18**	**<0**.**001**
Mean temp.	0.007	0.04	−0.09	0.08	0.853
Temp. seasonality	−0.05	0.07	−0.18	0.08	0.473
Mean prec.	0.01	0.05	−0.09	0.11	0.841
Prec. seasonality	0.02	0.03	−0.04	0.09	0.461
Bird diversity	−0.05	0.10	−0.25	0.15	0.643
**Sampling effort**	**0**.**87**	**0**.**05**	**0**.**78**	**0**.**96**	**<0**.**001**

## Discussion

Parasites can have profound ecosystem effects due to direct and indirect cascading effects on host population dynamics and interspecies interactions (Poulin, 1999; Lafferty *et al*., 2008; Dunne *et al*., 2013). Here, I demonstrated that haemosporidian diversity is ruled by host phylogenetic diversity, host body mass, and, for *Leucocytozoon*, host migratory status. More specifically, parasite diversity increases with increasing host phylogenetic diversity and heavier-bodied hosts. For *Leucocytozoon*, I also observed an increase in regional parasite diversity with an increasing proportion of migratory hosts. However, the influence of some of these variables varies according to the taxon of the parasite evaluated. Furthermore, temperature, precipitation and seasonality were not correlated with haemosporidian diversity in any model. In general, I showed that haemosporidian diversity was intrinsically associated with their host's diversity and body mass.

Parasites depend on their hosts to complete their life cycle. As a result, there is a strong relationship between host and parasite diversity. Previous research has shown that host taxonomic diversity is one of the main factors that drive parasite diversity (Hechinger and Lafferty, 2005; Kamiya *et al*., 2014*b*; Martins *et al*., 2020). However, host diversity alone might not paint the whole picture. Here, I show that host body mass plays an important role in determining the regional diversity of haemosporidian lineages. At the same time, since heavier-bodied avian hosts release more carbon dioxide, host body mass drives parasite diversity by representing a more attractive resource to vectors. Indeed, Filion *et al*. (2020) have also pointed out that host body mass is positively associated with regional *Plasmodium* prevalence. For *Leucocytozoon*, I observed that host migratory status enhances parasite diversity. It is possible that migrant hosts could contribute to parasite diversity by carrying their parasites through their flyways, increasing the odds of new parasite lineages colonizing that new region. Thus, the degree of connectivity among localities could be a potential driver of parasite diversification but might not play a role in all parasite-host systems.

Parasite diversity worldwide mirrors their host diversity (Poulin, 2014), however, the diversity of parasites at the host level is not constant. For example, host body mass is positively related to parasite diversity among most hosts and parasite taxa (Kamiya *et al*., 2014*a*). Indeed, I observed that, at a regional level, host body mass was related to parasite diversity in most models (except the *Plasmodium-only* model). Since larger hosts usually serve as hosts for more parasite species, the local pool of parasites inhabiting regions with large-sized hosts might be wider. Parasite diversity is also influenced by regional anthropogenic impacts. Previous research reported variation in haemosporidian composition and diversity among urban, polluted and deforested areas (Chasar *et al*., 2009; Ferreira *et al*., 2017; Fecchio *et al*., 2021*a*). However, the impact of anthropogenic factors on parasite diversity has not been uniform. While previous research has linked changes in parasite diversity with shifts in host composition, contrasting effects (positive, neutral and/or negative correlations) between urbanization/deforestation and parasite diversity have also been observed (Sehgal, 2015; Ferreira *et al*., 2017; Tchoumbou *et al*., 2020; Fecchio *et al*., 2021*a*). Overall, variation in spatial parasite diversity seems subject to more pressures than simply regional diversity of host species.

Furthermore, climatic conditions do not seem to influence haemosporidian diversity. Nonetheless, climatic conditions and seasonality can shape mosquito communities (Mayi *et al*., 2020) and increase parasite specificity (Fecchio *et al*., 2019). Changes in mosquito community composition and parasite specificity as a result of distinct patterns of temperature and precipitation may shape the composition of haemosporidians. For instance, de Angeli Dutra *et al*. (2023) demonstrated temperature variations were the main climatic driver of haemosporidian turnover. Therefore, climate should affect haemosporidian composition without enhancing parasite diversification. Nonetheless, due to data limitations, vector information could not be incorporated into the models. Moreover, Filion *et al*., 2020 have uncovered temperature seasonality as a major driver of *Plasmodium* prevalence, which is also coupled with parasite diversity (Van Hemert *et al*., 2019; Cuevas *et al*., 2020). Overall, climate might not affect parasite diversity, but only assemblage.

It is important to note that this research has limitations that must be acknowledged. Firstly, due to limitations on data, my analyses did not consider vector distribution, diversity or functional traits. Therefore, I could not account for the effects of vector biology on haemosporidian diversity. In addition, data on avian haemosporidians are very unevenly distributed worldwide, with the vast majority of the data being concentrated in the Americas and Europe. Indeed, most of Africa, Asia and Oceania continents have no data points. Sampling effort was the most influential predictor of haemosporidian diversity in all models. Even though sampling effort was used as a factor in our models, this study's results could still display a potential bias to reflect the conditions of regions with the greatest sampling effort.

In this study, I show that on a global spatial scale, host phylogenetic diversity and body mass were the main drivers of avian haemosporidian parasite diversity. I also showed that haemosporidian diversity increased in regions harbouring heavied-bodied host species. When haemosporidian genera were considered separately, I observed that *Leucocytozoon* diversity increased with higher proportions of migratory hosts. Furthermore, I found that climatic conditions had no effect on parasite diversity. Finally, I confirmed parasite diversity is intrinsically associated with their hosts' diversity.

## Supporting information

de Angeli Dutra supplementary material 1 de Angeli Dutra supplementary material

de Angeli Dutra supplementary material 2 de Angeli Dutra supplementary material

de Angeli Dutra supplementary material 3 de Angeli Dutra supplementary material

de Angeli Dutra supplementary material 4 de Angeli Dutra supplementary material

de Angeli Dutra supplementary material 5 de Angeli Dutra supplementary material

## Data Availability

Data that support the findings of this study are openly available in MalAvi (http://130.235.244.92/Malavi/), Open Traits datasets (https://opentraits.org/datasets.html) and as supplementary material for (Dufour et al., 2020). BirdLife International and Handbook of the Birds of the World (2020) Bird species distribution maps of the world, version 2020.1, can be accessed at http://datazone.birdlife.org/species/requestdis.

## Supplementary material

The supplementary material for this article can be found at https://doi.org/10.1017/S0031182024000313
