# Supplementary figures and images for: Assessing global drivers of parasite diversity: host diversity and body mass boost avian haemosporidian diversity
Source: Parasitology. 2024 Mar 7;151(5):478–84. doi: 10.1017/S0031182024000313 (PMC11106501; doi:10.1017/S0031182024000313)

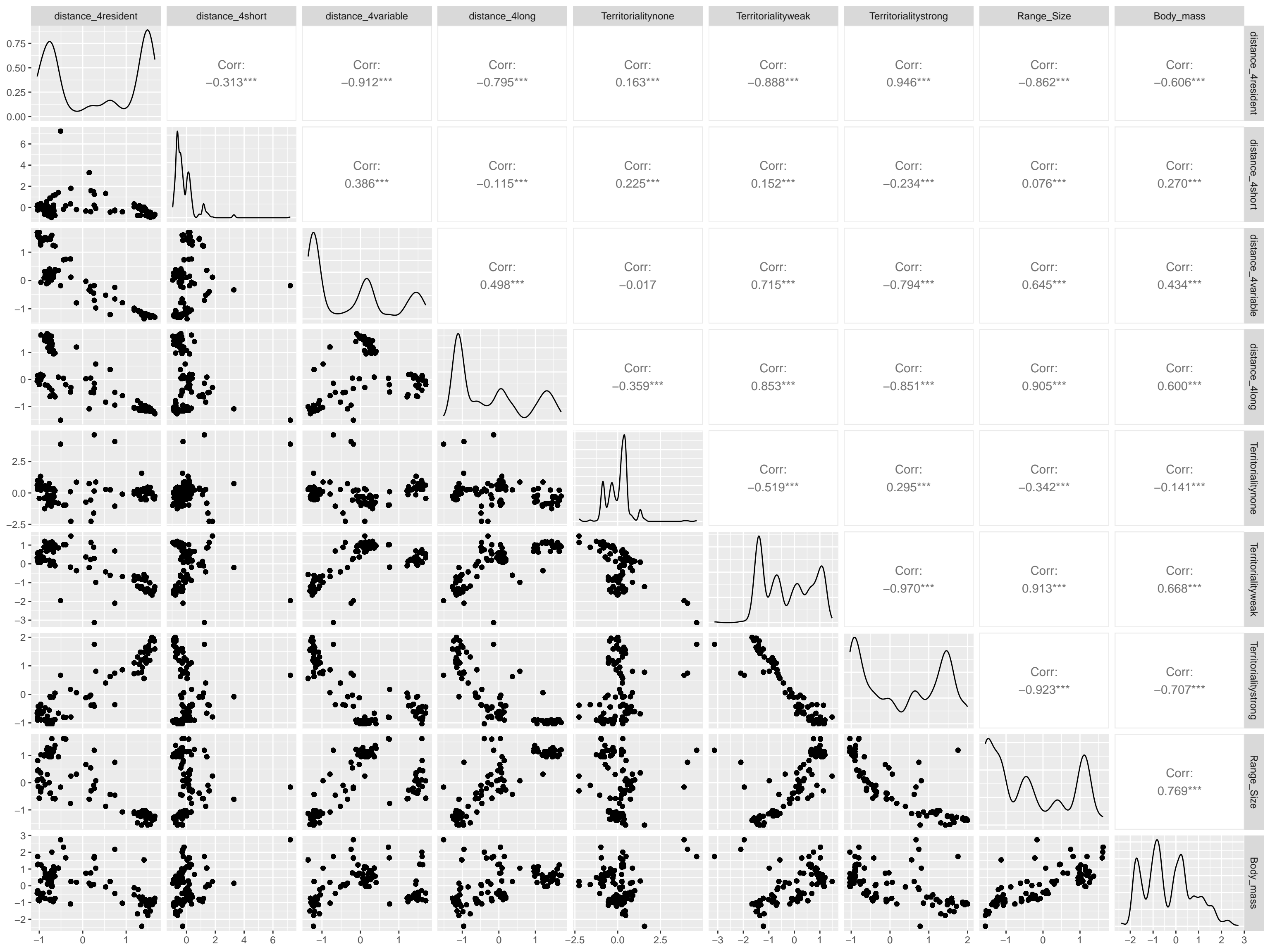

Supplement: de Angeli Dutra supplementary material 1 — de Angeli Dutra supplementary material [file S0031182024000313sup001.pdf]

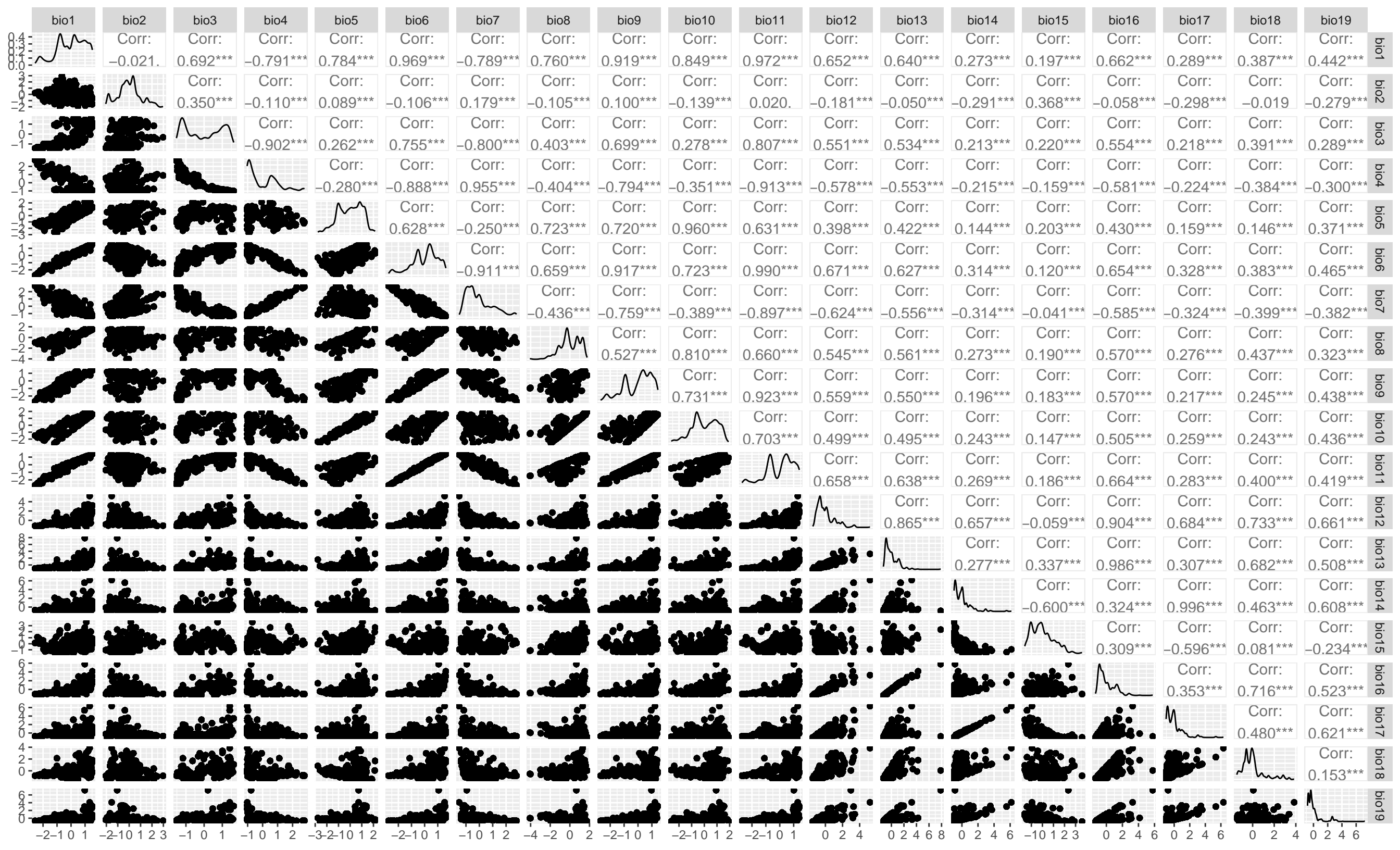

Supplement: de Angeli Dutra supplementary material 2 — de Angeli Dutra supplementary material [file S0031182024000313sup002.pdf]
