# Supplementary material for: Assessing global drivers of parasite diversity: host diversity and body mass boost avian haemosporidian diversity
Source: Parasitology. 2024 Mar 7;151(5):478–84. doi: 10.1017/S0031182024000313 (PMC11106501; doi:10.1017/S0031182024000313)
Supplement: de Angeli Dutra supplementary material 3 — de Angeli Dutra supplementary material [file S0031182024000313sup003.pdf]

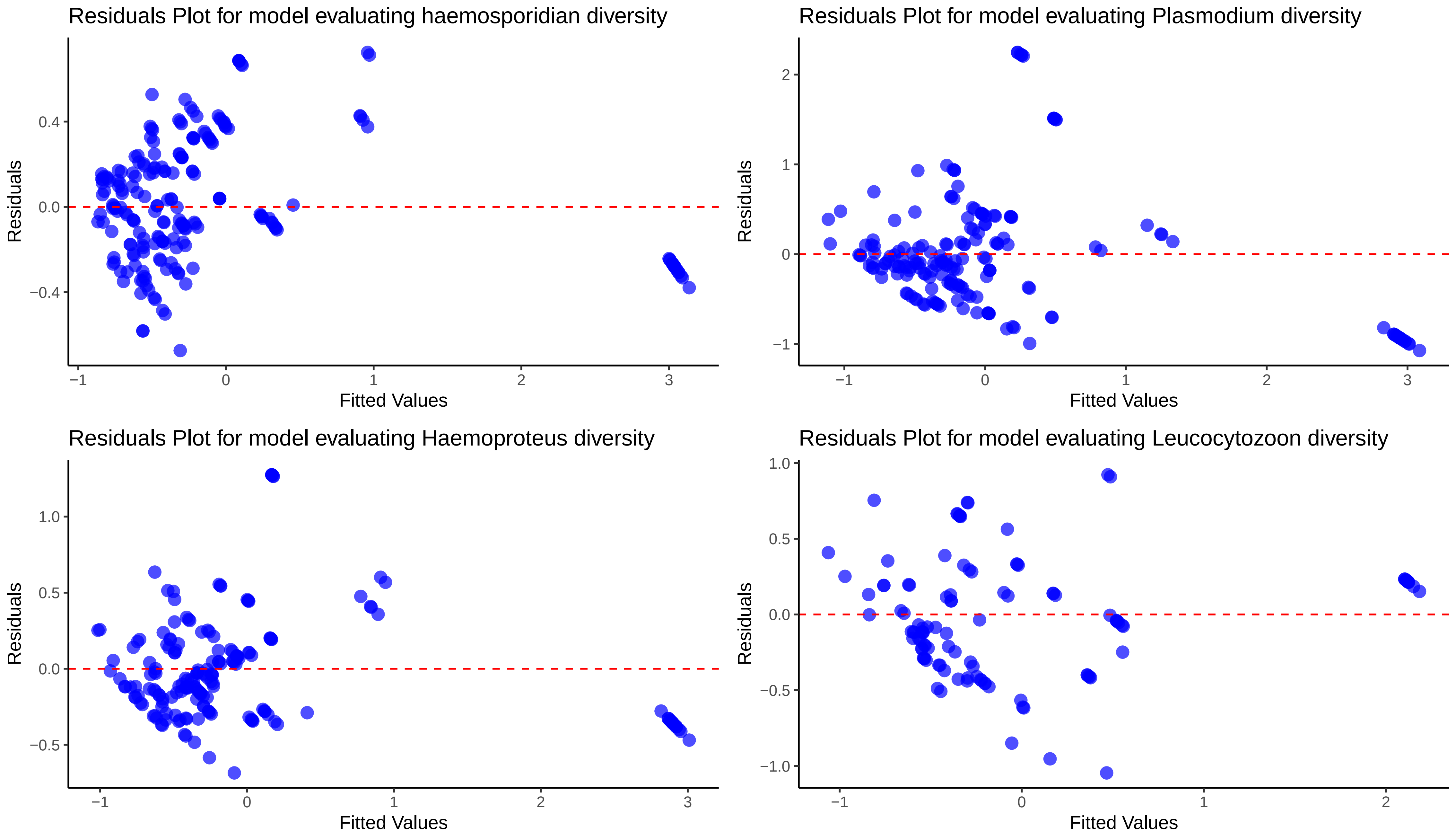

Residuals Plot for model evaluating haemosporidian diversity
Residuals Plot for model evaluating Plasmodium diversity
Residuals Plot for model evaluating Haemoproteus diversity
Residuals Plot for model evaluating Leucocytozoon diversity
Residuals
Fitted Values
0.4
0.0
−0.4
2
1
0
−1
1.0
0.5
0.0
−0.5
−1.0
3
